# Cellular cytokine and chemokine responses to parasite antigens and fungus and mite allergens in children co-infected with helminthes and protozoa parasites

**DOI:** 10.1186/s12950-015-0050-y

**Published:** 2015-01-20

**Authors:** Jana Hegewald, Richard G Gantin, Christian J Lechner, Xiangsheng Huang, Abram Agosssou, Yvon F Agbeko, Peter T Soboslay, Carsten Köhler

**Affiliations:** Institute for Tropical Medicine, University Clinics Tübingen, Wilhelmstraße 27, 72074 Tübingen, Germany; Institut National d’Hygiène - Onchocerciasis Reference Laboratory, Sokodé, Togo; Centre Hospitalier Régional, Service Pédiatrie, Sokodé, Togo

**Keywords:** Schistosomiasis, Amoebiasis, Hookworm, Co-infection, Cytokine, Chemokine, Allergen, Skin prick test

## Abstract

**Background:**

In sub-Saharan Africa poly-parasite infections are frequently observed in children, and with poly-parasitism modulating immune mechanisms, mediated by cytokines and chemokines, are required to prevent overwhelming inflammation and host tissue damage. We analyzed in children co-infected with helminthes and protozoan parasites their cellular production of regulatory and pro-inflammatory cytokines and chemokines in response to parasite antigens and allergens.

**Methods:**

Intestinal and intravascular parasite infections were detected in stool and urines samples. The *in vitro* cellular cytokine and chemokine responses of peripheral blood mononuclear cells (PBMC) to parasite antigens and allergens were analysed in children (n = 87) with single and poly-parasite infection, and skin prick test reactivity to fungus and mite allergens was determined in singly and poly-parasitized children (n = 509).

**Results:**

In children *Entamoeba histolytica/dispar* (62%), *Necator americanus* (31%), *Schistosoma haematobium* (28%), *S. mansoni* (21%), *Hymenolepis nana* (2%) and *Strongyloides stercoralis* (1%) were diagnosed. Singly infected were 37%, 47% were positive for 2 or more parasite species and 16% were infection-free. When PBMC were stimulated *in vitro* with parasite antigens and allergens, regulatory-type cytokine IL-27 and alarmin-type IL-33 enhanced with poly-parasite infections whilst IL-10 and pro-inflammatory MIP3-α/CCL20 and MIG/CXCL9 were produced in similar amounts in singly or poly-parasitized children. The co-stimulation *in vitro* of PBMC with mite allergens and *Ascaris lumbricoides* antigens depressed the allergen-induced pro-inflammatory IL-27, IL-33 and MIP3-α/CCL20 responses while regulatory IL-10 remained unaffected. Post albendazole and/or praziquantel treatment, the cellular release of IL-10, IL-33, MIP3-α/CCL20 and MIG/CXCL9 lessened significantly in all children infection groups. Skin prick test (SPT) reactivity to fungus *Aspergillus fumigatus* and mite *Dermatophagoides pteronyssinus* allergens was investigated in 509 children, and positive SPT responses were found in 23% of the infection-free, and in 47%, 53% and 56% of the singly, doubly and poly-parasite infected, respectively.

**Conclusions:**

In children co-infected with helminthes and protozoan parasites a mixed cellular response profile of both inflammatory and regulatory chemokines and cytokines was stimulated by individual antigens and allergens, pro-inflammatory cytokines and chemokines enhanced with an increasing number of parasite infections, and in poly-parasitized children skin prick test reactivity to allergens extracts was highest.

## Background

Despite the frequent occurrence of multiple parasite infections [1], little is known how concurrent parasite infections influence immune responsiveness in patients and few studies addressed the impact of helminth and intestinal protozoa co-infections on immune responses in children. The intensity and prevalence of helminth parasite infections is age-dependent with children being often the most affected [2,3], and poly-parasitism being more frequent than single parasite infections [1,4,5]. The existing epidemiological surveys on helminth co-infections in children indicate positive associations between schistosomes and soil transmitted helminths (STH) [5] which means high prevalence of mixed infections, as well as higher intensities of infection in co-infected patients [6]. For multiply infected patients, additive effects on the cellular reactivity and the down-modulation of cytokines are proposed [6]. In human mono-infections with STH an increased secretion of Th2-type cytokines associated with decreased cellular proliferation to specific parasite antigens and mitogens has been observed [7,8]. Protective Th2-type associated mechanisms have been suggested which may act distinctly during parasite migration leading to a reduction in numbers, size, and motility of migratory larvae [9-11] mediating a partial protection which will prevent an endless accumulation of adult helminthes parasites in the host [6]. In situations where several parasite species co-exist, where balanced and to some extent compromised immune responses are required, regulatory T cells and regulatory cytokines like IL-10 and IL-27 could represent the key components to prevent overwhelming inflammation and tissue destruction, but multiple and chronic infections may also drive immune responsiveness towards exhaustion. In poly-parasitized adults, parasite-specific cellular reactivity was significantly reduced in doubly infected individuals than in patients with a single filarial infection, and anti-parasite treatment greatly changed their cytokine and chemokine responses [9,12,13]. Generally accepted, Th2 type cytokines and chemokines play an essential role in the pathogenesis of allergic inflammation. Th2-type cytokines like IL-4, IL-5, IL-9 and IL-13 contribute to the pathophysiological conditions of allergy and asthma, and chemokines like eotaxin/CCL11, RANTES/CCL5 and monocyte chemoattractant proteins (MCPs) contribute decisively to the recruitment of basophil and eosinophil granulocytes as well as mast cells in tissues of allergic inflammation [14]. Chemokines attract Th2-type cells into bronchi and gut mucosal tissues in response to allergen exposure or intestinal helminthes parasite infection [15,16]. Intravascular parasite infections were described to ameliorate and even prevented allergen induced skin sensitization in humans [17] as well as airway hyper-reactivity in experimental animal studies [18]. Such an immune modulating capacities of pathogens has been extended to situations without active infection where exposure to environmental non-viable microbial products sufficed to reduce the occurrence of hay fever, atopic asthma, and atopic sensitization to environmental allergens [19] In children, poly-parasite infections with *S. haematobium/S. mansoni, E. histolytica/E. dispar*, and *N. americanus* generated prominent pro-inflammatory cytokine and chemokine responses, and anti-helminth treatment lessened inflammatory chemokine responses whilst the Th2 responsiveness in co-infected children increased [5]. Furthermore, long-term periodic anti-helminth treatments were associated with an increased prevalence of allergen skin test reactivity [20] and eczema symptoms [21].

In the present work, we analyzed in children infected with helminthes and protozoan parasites the cellular responses to protozoa or helminthes extracts and allergens, and observed distinct cytokine and chemokine production profiles; furthermore, in co-infected children prominent inflammatory chemokine and cytokine responses were observed together with enhanced allergen skin test reactivity.

## Methods

### Study participants

This study was conducted in the central region of Togo, Africa. All children examined were attending primary public schools in suburban quarters of the town of Sokodé. For stool and urine examinations, 50-mL polypropylene tubes were distributed to the pupils and collected the next morning; diagnostic procedures were performed by the laboratory staff at the Centre Hospitalier Régional (CHR), Sokodé.

On the basis of the diagnostic results 4 infection groups were formed. All children who did not have a parasite infection and were apparently healthy (no fever, headache, vomiting, abdominal pain, diarrhea, dizziness, or skin lesions) were considered to be control children (Group G0 = NEG). Children with single infection Group G1, those with double infection Group G2 and with three and parasite infections are Group G3+.

In Tables 1 and 2 the children infection groups G0, G1, G2 and G3+ are listed from whom peripheral blood mononuclear cells (PBMC) were isolated (n = 87) and studied *in vitro* for cytokine and chemokine responses to parasite antigens and fungus and mite allergens. Demographic data and infection details from participating children are shown in Tables 1 and 2; the infection groups G0, G1, G2 and G3+ are listed by parasite species and abbreviations are indicated: Na = Necator americanus; Eh = Entamoeba histolytica/dispar; Gl = Giardia lamblia; Hn = Hymenolepis nana; Sh = Schistosoma haematobium; Sm = Schistosoma mansoni; Ti-Trichomonas intestinalis. Children from whom PBMC were isolated were invited with their parents or legal guardians to the CHR Sokodé for blood sample collection. Children who showed signs of malaria (thick blood smears positive for *Plasmodium* species and fever), or who had diarrhea were excluded from the study. None of the children presented with *E. histolytica* trophozoites containing ingested red blood cells in stool samples, bloody stool, or clinical signs of invasive amoebiasis. Eight weeks after treatment, stool, urine, and blood samples from G0, G1, and G3+ children (*n =* 22) were re-examined in the same fashion as before treatment.

**Table 1:** **Demographic data of children from whom peripheral blood mononuclear cells (PBMC) were isolated and PBMC used in cell culture experiments for the evaluation of cytokine and chemokine responses to parasite antigens and fungus and mite allergens**

		**n**	**Median age**	**Min age**	**Max age**
G0 = NEG (14)	f	8	11	10	12
	m	6	11	10	12
G1 (32)	f	15	10	9	13
	m	17	12	10	13
G2 (18)	f	5	11	9	12
	m	13	11	10	13
G3+ (23)	f	4	12	11	12
	m	19	11	10	13

Children were grouped according to the presence or absence of parasite infections; without parasite infection (Group G0 = NEG), children with single infection (Group G1), with double (Group G2) and with poly-parasite infections (Group G3+). From children grouped by number of parasite infection (Abbreviations: f/m = female/male).

**Table 2:** **Infections listed by parasite species in children from whom peripheral blood mononuclear cells (PBMC) were isolated and PBMC studied** ***in vitro*** **for cytokine and chemokine responses to parasite antigens and fungus and mite allergens**

G0	NEG									14	NEG = 16%
G1									Ss	1	
G1							Ti			1	
G1					Sm					1	
G1				Sh						4	
G1			Na							4	
G1		Eh								21	G1 = 37%
G2						Gl		Hn		1	
G2				Sh	Sm					1	
G2			Na			Gl				2	
G2			Na	Sh						2	
G2		Eh					Ti			1	
G2		Eh				Gl				4	
G2		Eh			Sm					2	
G2		Eh		Sh						1	
G2		Eh	Na							4	G2 = 21%
G3+			Na	Sh		Gl				1	
G3+			Na	Sh	Sm					1	
G3+		Eh				Gl	Ti			1	
G3+		Eh		Sh	Sm					7	
G3+		Eh	Na				Ti			1	
G3+		Eh	Na				Ti	Hn		1	
G3+		Eh	Na			Gl	Ti			1	
G3+		Eh	Na		Sm					3	
G3+		Eh	Na	Sh						4	
G3+		Eh	Na	Sh	Sm					2	
G3+		Eh	Na	Sh	Sm		Ti			1	G3 = 26%
										87	
Infected (n)		54	27	24	18	10	7	2	1		
Infection (%)		62	31	28	21	11	8	2	1		

Children without infection are Group G0 = NEG, children with single infection Group G1, those with double infection Group G2 and with three and parasite infections are Group G3+. Infections are listed by parasite species and abbreviations are shown: Na = Necator americanus; Eh = Entamoeba histolytica/dispar; Gl = Giardia lamblia; Hn = Hymenolepis nana; Sh = Schistosoma haematobium; Sm = Schistosoma mansoni; Ti-Trichomonas intestinalis).

In Tables 3 and 4 the children infection groups G0, G1, G2 and G3+ are listed in whom skin prick test reactivity was evaluated (n = 509); demographic data and infection details from participating children are shown.

**Table 3:** **Demographic data (age, sex) of children evaluated for skin prick test reactivity**

		**n**	**Median age**	**Min**	**Max**
G0 = NEG (287)	f	121	11	7	12
	m	166	11	9	12
G1 (137)	f	51	11	8	12
	m	86	11	8	12
G2 (58)	f	25	12	9	12
	m	33	12	9	12
G3+ (27)	f	9	12	11	12
	m	18	11	10	12

Children were grouped according to the presence or absence of parasite infections: without parasite infection (Group G0 = NEG), with single infection (Group G1), double (Group G2) and with poly-parasite infections (Group G3+).

**Table 4:** **Parasite infections in children evaluated for skin prick test reactivity**

	G0 = NEG								287	G0 = 56.4%
	G1							Hn	7	
	G1						Ti		5	
	G1					Gl			1	
	G1				Na				43	
	G1			Sm					12	
	G1		Sh						18	
	G1	Eh							51	G1 = 26.9%
	G2					Gl	Ti		2	
	G2				Na			Hn	1	
	G2				Na		Ti		3	
	G2				Na	Gl			1	
	G2			Sm	Na				3	
	G2		Sh				Ti		1	
	G2		Sh		Na				6	
	G2		Sh	Sm					3	
	G2	Eh						Hn	1	
	G2	Eh					Ti		12	
	G2	Eh				Gl			6	
	G2	Eh			Na				10	
	G2	Eh		Sm					1	
	G2	Eh	Sh						8	G2 = 11.4%
	G3+			Sm	Na	Gl			1	
	G3+		Sh			Gl	Ti		1	
	G3+		Sh		Na		Ti		1	
	G3+		Sh	Sm				Hn	1	
	G3+	Eh					Ti	Hn	2	
	G3+	Eh				Gl	Ti		3	
	G3+	Eh			Na		Ti		3	
	G3+	Eh		Sm			Ti		2	
	G3+	Eh		Sm	Na				2	
	G3+	Eh		Sm	Na		Ti		1	
	G3+	Eh		Sm	Na	Gl	Ti		1	
	G3+	Eh	Sh				Ti		1	
	G3+	Eh	Sh			Gl			1	
	G3+	Eh	Sh		Na				4	
	G3+	Eh	Sh	Sm					2	
	G3+	Eh	Sh	Sm	Na		Ti		1	G3+ = 5.3%
									509	
Infeted(n)		112	48	30	81	17	39	12		
Infection (%)		22	9	6	16	3	8	2		

Children were grouped according to presence or absence of infection and by parasite species. Without infection were Group G0 = NEG, children with single infection were Group G1, with double infection were Group G2 and with three and more parasite infections were Group G3+. Infections are listed by parasite species and abbreviations are shown: Na = Necator americanus; Eh = Entamoeba histolytica/dispar; Gl = Giardia lamblia; Hn = Hymenolepis nana; Sh = Schistosoma haematobium; Sm = Schistosoma mansoni; Ti-Trichomonas intestinalis.

All children examined received a single dose of albendazole (400 mg), and those infected with *S. haematobium* or *S. mansoni* were treated with praziquantel according to the guidelines of the Togolese Ministry of Health. Diagnostic procedures to distinguish *E. histolytica* from *E. dispar* are not available at the laboratory facilities at the CHR, Sokodé, and treatment of *E. histolytica/E. dispar* is recommended on evidence of invasive amoebiasis (i.e., *E. histolytica* trophozoites containing ingested red blood cells in stool samples, bloody stool, or clinical signs of invasive amoebiasis).

Authorization and approval for the present work was granted by the Togolese Ministry of Health (292/99/MS/CAB and 0407/2007/MMS/CAB/DGS), the regional ministry of education (MENR/SG/DRERC/13.06.2001), the regional health authority (MS/DGS/DRS/RC/No.220 and MS/DGS/DRS/RC/No.261) and the Ethik Kommission at University Clinics Tübingen/Germany (No. 188/2008/BO2). Oral informed consent was given by all participating children, and written consent was provided by all parents or legal guardians after thorough explanation of the procedures, aims, and risks of the study; moreover, to ensure informed understanding, explanations were always given in the local language by the medical staff at the CHR, Sokodé.

### Parasitological analysis

For determination of intestinal helminth and protozoan infections, fresh stool samples (0.5 g) were mixed with saline and dispersed on 2 microscope slides covered with a 24×48-mm slide; samples were examined by laboratory technicians. All stool samples were examined using the Kato-Katz technique for quantification of helminth eggs per gram of stool (helm-TEST; Labmaster). To detect *S. haematobium* infection, 10 ml of urine from each participant was filtered (polycarbonate membrane; pore size, 12 μm; Whatman); the filters were then examined under a microscope, and *S. haematobium* eggs were quantified. Thick blood smears were analyzed for the presence of *Plasmodium* species before and 8 weeks after anti parasite treatment of G0, G1, and G3+ children.

### Skin prick test examination

*Aspergillus fumigates* and *Dermatophagoides pteronysinus* prick test solutions (Bencard Allergie GmbH, München, Germany) were used. Allergens and positive histamine and negative saline controls were pricked onto the volar surface of the forearm, and reactions were recorded after 15 min. A skin prick test (SPT) weal cut-off diameters of 3 mm or larger was considered as a positive reaction. All tests were conducted by the same observer (RGG); reactions below this level were considered as non-specific and not reproducible.

### Isolation of peripheral blood mononuclear cells (PBMCs) and cell culture experiments

Venous blood (9 ml) was collected from participating children before and 8 weeks after anti-parasite treatments. PBMCs were isolated by ficoll-hypaque density gradient centrifugation at 340 *g* for 35 min at room temperature. Plasma samples were collected and frozen at −20°C until further use. PBMCs were cultured in RPMI 1640 medium (Gibco) supplemented with 0.025 mol/l HEPES buffer, 100 U/ml penicillin, 100 μg/ml streptomycin, and 0.25 μg/ml amphotericin B (Sigma). The cell suspension was adjusted to 2,5 × 10^6^ cells/ml and 1,25 × 10^6^ PBMC were cultured in 0.5 ml RPMI 1640 supplemented as described above plus 10% heat-inactivated fetal calf serum (Biochrom). PBMCs were cultured in 48-well plates at 37°C in 5% CO2 with saturated humidity in the presence of either *Entamoeba histolytica* strain HM1 antigen (EhAg; 10 μg/ml), *Plasmodium falciparum* schizont lysate (PfAg; 1 × 10^8^ schizonts/ml), *Schistosoma mansoni* adult worm extract (SmAg; 10 μg/ml), *Echinococcus multilocularis* metacestode extract (EmAg; 30 μg/ml), *Ascaris lumbricoides* adult worm extract (AscAg; 5 μg/ml)*,* Lipopolysaccharid from *Escherichia coli* 026:B6 (LPS; 10 μg/ml), *Dermatophagoides pteronyssinus* (Dp, 20 μg/ml)*, Dermatophagoides farinae* (Df, 20 μg/ml), *Aspergillus fumigatus* (Af; 20 μg/ml), *Candida albicans* (Ca; 20 μg/ml) (Dp, Df, Af, and Ca extracts were all from Allergopharma, Reinbek, Germany), or PBMC were left unstimulated (baseline). Cell cultures were terminated after 48 hours, and cell-free supernatants were collected and stored below −20°C until further use.

### Determination of cytokine and chemokine production in cell culture supernatants

Quantitative enzyme-linked immune sorbent assay (ELISA) was performed with commercially available assays to determine in cell culture supernatants the levels of the cytokines IL-10, IL-27 and IL-33, as well as of the chemokines MIP3-α/CCL20 and MIG/CXCL19 (Duo-Set; R&D Minneapolis, MN, USA). Sample concentrations of each cytokine and chemokine were quantified from standard curves generated with recombinant chemokines/cytokines, and the lower limit for their detection was 30 pg/ml for IL-10, 170 pg/ml for IL-27, 25 pg/ml for IL-33, 15 pg/ml for MIP3-α/CCL20 and 60 pg/ml for MIG/CXCL9. ELISAs were performed as recommended by the manufacturer.

### Statistical analysis

JMP software (versions 5.1; SAS Institute) was used for statistical analysis of data. Because of multiple comparisons, the level of significance was adjusted according to Bonferroni–Holm (alpha = 0.0018). For the cytokine and chemokine analyses, differences between groups were determined after logarithmic transformation to stabilize the variance of data (log [pg/ml + 1]). Paired data from patients were evaluated by *t*-test and unpaired data of patient groups were compared using Wilcoxon’s rank sum test.

## Results

### Parasite infections in children

For *in vitro* analyses of cellular responses in children, peripheral blood, stool and urine samples were collected and examined from 87 children (Tables 1 and 2). With 62% (n = 54) *Entamoeba histolytica/dispar* was the most frequent parasite infection diagnosed, followed by hookworm (31%, n = 27) while 28% (n = 24) and 21% (n = 18) were positive for *Schistosoma haematobium* or *S. mansoni,* and 16% (n = 14) were parasite infection-free. *Giardia lamblia* infection was present in 11% (n = 10), *Hymenolepis nana* eggs in 2% (n = 2) and *Strongyloides stercoralis* larvae were found in n = 1 (1%) of the participants. Singly infected were 37% (n = 32), 21% of the children were positive for 2 (n = 18) and 26% (n = 23) positive for 3 and more parasite species (Table 2).

### Cellular cytokine and chemokine production of IL-10, IL-27, IL-33, MIP3-α/CCL20 and MIG/CXCL9 to parasite antigens and allergens

**Figure 1:**
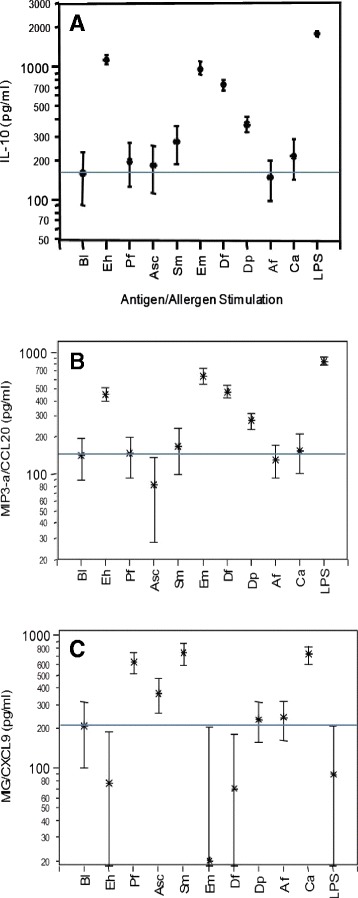
**The inducible and spontaneous cellular production (in pg/ml) of interleukin 10 (IL-10) (Part A), Monocyte Inflammatory Protein 3-α (MIP-3α/CCL20) (Part B) and Monokine Inducible by Interferon-γ (MIG/CXCL9) (Part C) by peripheral blood mononuclear cells (PBMC) from children is shown.** Cellular cytokine or chemokine production was quantified by specific ELISA in cell culture supernatants after stimulation of PBMC for 48 hours with parasite-specific antigens from *Entamoeba histolytica* (Eh), *Plasmodium falciparum* (Pf), *Ascaris lumbricoides* (Asc), *Schistosoma mansoni* (Sm), *Echinococcus multilocularis* (Em), bacterial lipopolysaccharid (LPS) from *Escherichia coli* or allergen extracts from *Dermatophagoides farinae* (Df), *Dermatophagoides pteronyssinus* (Dp), *Aspergillus fumigatus* (Af) and *Candida albicans* (Ca). The spontaneous cytokine or chemokine releases by PBMC, i.e. cell cultures without antigen or allergen activation, are shown as baseline (Bl), and the horizontal line indicates the mean spontaneous cytokine or chemokine release. Cellular production of IL-10, MIP-3α/CCL20 and MIG/CXCL9 are shown as means with the 95% upper and lower confidence interval.

The cellular production of cytokines and chemokines in children was selectively inducible by parasite antigens and allergen extracts. Antigens of the intestinal protozoan *Entamoeba histolytica* (EhAg)*,* cestode *Echinococcus multilocularis* (EmAg)*,* mite-derived *Dermatophagoides spp.* (Dp, Df) and bacterial LPS were strong inducers of cellular IL-10 and MIP3-α/CCL20 release (Figure 1). In contrast, nematode *Ascaris-,* trematode *Schistosoma-* and protozoa *Plasmodium*-derived antigens and yeast *Candida albicans* (Ca) extracts selectively induced MIG/CXCL9, but did not activate IL-10 production by PBMC above the spontaneously released baseline levels (Figure 1). The cestode *Echinococcus* antigens activated IL-27 (p < 0.04) whilst *Ascaris* extracts suppressed cellular IL-27 responses below (p < 0.002) the spontaneous release (baseline), and in opposite, *Ascaris* extracts enhances enhanced IL-33 (p = 0.006) above baseline levels and *Echinococcus* antigens suppressed IL-33 (p = 0.03) cellular release (data not shown).

### Cytokine and chemokine responses in singly and poly parasitized children

**Figure 2:**
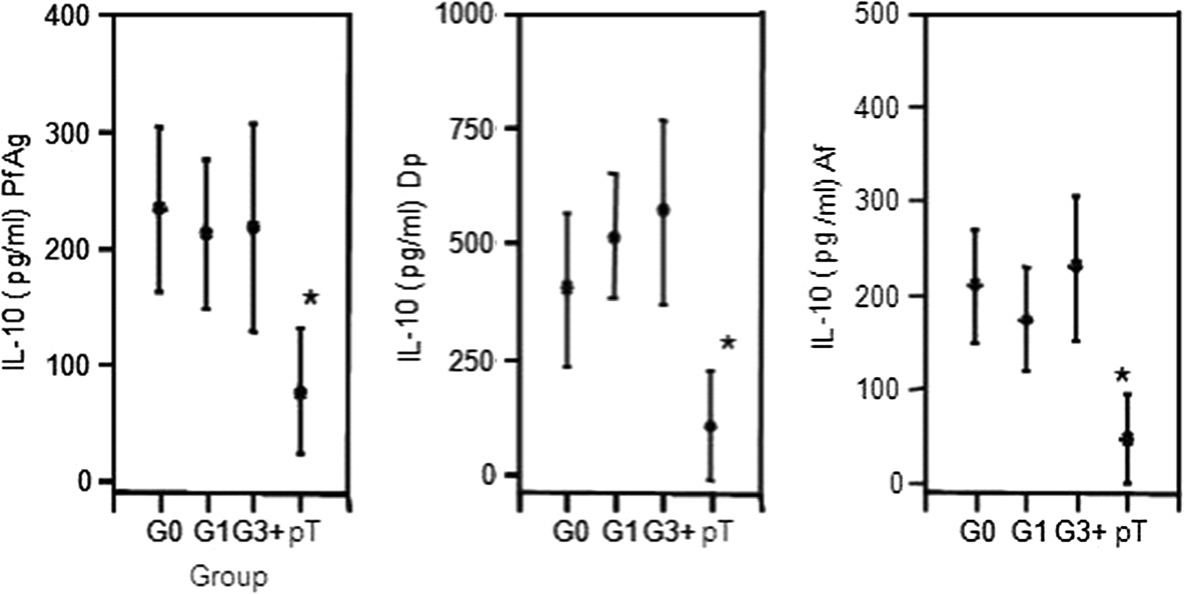
**The cellular production of interleukin 10 (IL-10) by peripheral blood mononuclear cells (PBMC) is shown in children without parasite infection (Group G0, n = 11), children with single infection (Group G1, n = 14) and children with poly-parasite infections (Group G3+, n = 7), and also, IL-10 production in children at 8 weeks post anti-parasite treatment (pT, n = 19) is given.** The release of IL-10 by PBMC was quantified by cytokine specific ELISA in cell culture supernatant of PBMC activated with antigens from *P. falciparum* (Pf), with *D. pteronyssinus* (Dp) or with *A. fumigatus* (Af) is shown in G0, G1, G3+ children before and 8 weeks post anti-parasite treatment (pT). Cellular production of IL-10 is shown as means with the 95% upper and lower confidence interval. The level of significance was adjusted according to Bonferroni–Holm, and significant differences between groups are indicated as *P ≤ 0.002.

**Figure 3:**
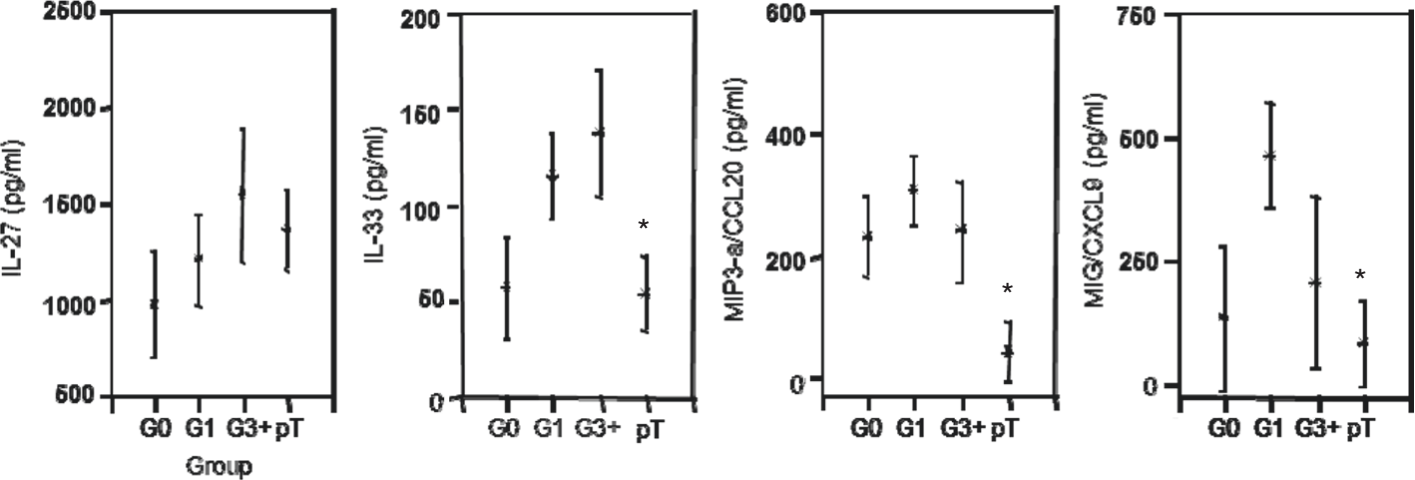
**The cellular release of IL-27, IL-33, MIP3-a/CCL20 and MIG/CXCL9 in response to allergen extracts of** ***Dermatophagoides pteronyssinus*** **(Dp) and** ***Aspergillus fumigatus*** **(Af), in singly and poly-parasitized children; children without parasite infection (Group G0, n = 11), children with single infection (Group G1, n = 14) and children with poly-parasite infections (Group G3+, n = 7), and the cytokine and chemokine production in children at 8 weeks post anti-parasite treatment (pT, n = 19) is shown.** The cytokine and chemokine responses to the extracts of *D. pteronyssinus* (Dp) and *A. fumigatus* (Af) are merged. The cellular productions of IL-10, IL-27, MIP-3α/CCL20 and MIG/CXCL9 are shown as means with the 95% upper and lower confidence interval. The level of significance was adjusted according to Bonferroni–Holm, and significant differences between groups are indicated as *P ≤ 0.002.

In children with single and multiple parasite infections, the IL-10 responses to antigens and allergens were similarly expressed irrespective of the number and species of parasite infections, i.e. the levels of IL-10 as released by PBMC in pg/ml from singly infected children did not further enhance with concurrent and additional parasite challenge (Figure 2). Following anti-parasite treatment, and without having achieved complete parasite elimination, the cellular production of IL-10 significantly diminished in the children (Figure 2). In the children infection groups, the amounts of IL-27 and IL-33 as produced by their PBMC increased in order G0 < G1 < G3+; the IL-27 production remained unchanged post anti-parasite treatment (pT), whilst the cellular IL-33 production returned pT to the lowest levels as found in G0 children (Figure 3). Irrespective of the children infection groups G0, G1, G3+, the release of MIP3-α/CCL20 remained at similar levels when PBMC were activated with *D. pteronyssinus* (Dp) and *A. fumigatus* (Af) (Figure 3). At 8 weeks post anti-parasite treatment (pT), the MIP3-α/CCL20 significantly diminished. For MIG/CXCL9, cellular release was low in G0 children, enhanced in G1 and G3+ children to some extent, and at 8 weeks post anti-parasite treatment (pT), it lessened to levels as found in the G0 children group (Figure 3).

### Cytokine and chemokine release with antigen and allergen co-stimulation

**Figure 4:**
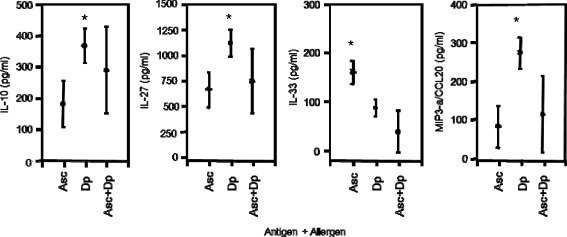
**Cellular release (in pg/ml) of IL-10, IL-27, IL-33 and MIP3-a/CCL20 by PBMC activated alone with helminth-derived** ***Ascaris lumbricoides*** **(Asc) or allergen extracts from** ***Dermatophagoides pteronyssinus*** **(Dp) or after co-activation with Asc antigen and DP allergen.** PBMC were isolated from singly and poly-parasitized children and cultured in vitro for 48 hours. Cellular production of IL-10, IL-27, IL-33, MIP-3α/CCL20 and MIG/CXCL9 are shown as means with the 95% upper and lower confidence interval. The level of significance was adjusted according to Bonferroni–Holm, and significant differences between groups are indicated as *P ≤ 0.002.

In order to evaluate what effect simultaneous antigen and allergen exposure might exert on cellular cytokine and chemokine production, PBMC from G0, G1 and G3+ children were activated with allergen extracts and then exposed to helminth or protozoa antigens for 72 hours. While mite allergen extract strongly activated IL-10, IL-27 and MIP3-α/CCL20 production, IL-33 became suppressed (Figure 4). The co-culture of allergen-stimulated PBMC with helminth *Ascaris* antigens lessened the production of IL-27, IL-33 and MIP3-α/CCL20 to levels as observed with *Ascaris* antigens exposure alone; the IL-10 production as induced by *D. pteronyssinus* (Dp) alone did not diminish in the presence of helminth antigen.

Exposure of PBMC to *Entamoeba histolytica* extract activated IL-10 above those levels as induced by *D. pteronyssinus* (Dp) alone (Figure 1), and the simultaneous exposure to both extracts further enhanced cellular IL-10 production. Protozoan *E. histolytica* antigen and the co-activation with mite allergens did not affect IL-27 and MIP3-α/CCL20 production by PBMC (not shown). Noteworthy, co-exposure of cells to *Ascaris* antigen in the presence of *D. pteronyssinus* (Dp) allergen extracts suppressed the release of IL-33 by PBMC (Figure 4).

### Skin prick test responses to allergens in singly and parasite co-infected children

**Figure 5:**
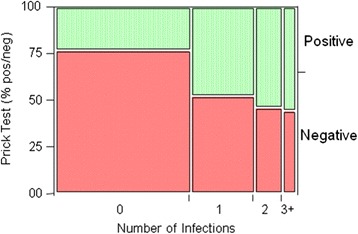
**Skin prick test reactivity (positive/negative) in children (total n = 509) to** ***Aspergillus fumigates*** **and** ***Dermatophagoides pteronysinus*** **prick test solutions were evaluated in n = 287 non-infected (G0), in n = 137 singly (G1), in n = 58 doubly (G2) and n = 27 poly-parasitized (G3+) children.** Allergens and positive histamine and negative saline controls were pricked onto the volar surface of the forearm, and reactions were recorded after 15 min. A skin prick test (SPT) weal cut-off diameters of 3 mm or larger was considered as a positive reaction. (p < 0.0001 between G0 and G1).

For analysis of skin prick test (SPT) responsiveness in singly, doubly and poly-parasitized children, stool and urine samples were collected and examined from n = 509 pupils (Tables 3 and 4). Skin prick test responses to *Aspergillus fumigatus* (Af) and *Dermatophagoides spp.* (Df) were evaluated in n = 287 non-infected, in n = 137 singly, in n = 58 doubly and n = 27 poly-parasitized children (Table 4). In non-infected children skin prick test responses developed less often (p < 0.0001) than in those with parasite infections (Figure 5). In non-infected children 23%, in singly infected 47%, in doubly parasite-infected 53% and those with 3 and more parasite infections 56% developed positive SPT reactions to Af and Df extracts*.* Notable, the presence or absence of *Entamoeba histolytica/dispar* or hookworm infections did not interact with SPT responses while *Schistosoma spp.* infections were associated with an enhanced SPT reactivity to Af and Df extracts (p = 0.007).

## Discussion

The present study revealed in children infected with helminthes and protozoan parasites distinctive cellular cytokine and chemokine response profiles to antigens of protozoa, helminthes, bacteria and allergens. The intestinal protozoa *Entamoeba histolytica,* bacterial LPS and mite-allergens stimulated the production of the regulatory cytokine IL-10 and monocyte-inflammatory MIP3-α/CCL20. The *Plasmodium*, helminthes and *Candida* extracts strongly induced the cellular release of MIG/CXCL9, a monokine inducible by IFN-γ, while pro-inflammatory IL-33 was inducible by *Ascaris* and *Echinococcus* antigens only.

The IL-10 production as observed in infection-free children (group G0) did not further enhance with double (G2) or poly-parasite (G3+) infections, however, it diminished post anti-parasite treatment. This we have similarly observed in hookworm, filaria and *E. histolytica* co-infected adults [12]. The regulatory cytokine IL-10 will modulate Th1-type responses to antigens by depressing pro-inflammatory TNF, IFN-γ and IL-12p70 [22] contributing to active and general immune suppression and preventing inflammatory disease manifestations [23-25], but elevated IL-10 production levels may also facilitate the persistence of pathogens. Concurrent infections with several parasite species may counteract the regulatory effects of IL-10, notably when pathogens enhance the production of inflammatory cytokines and chemokines [26], and therefore, not singular cytokines but rather distinct cytokine response profiles may define the expression of immunity and severity of disease [27].

As a cytokine with regulatory capacity, IL-27 will initiate IFN-γ responses and promote IL-10 synthesis by regulatory T cells, then attenuate Th2 and Th17 cells [28] and depress pro-inflammatory cytokines and chemokines [29,30]. In children, the production levels of regulatory IL-27 and also of inflammatory IL-33 rose with the number of infections (G0 < G < G3+) and these responses were inducible by mite-allergen extracts. IL-27 was shown to be a key regulator of IL-10 and IL-17 production by human CD4+ T cells thus providing a dual regulatory mechanism to control autoimmunity and tissue inflammation [28-31]. The cytokine IL-33 was suggested to function as an “alarmin” [32]; in infants with severe malaria IL-33 levels were found enhanced, IL-33 concentrations in plasma correlated positively with parasite densities, and IL-33 diminished strongly following *P. falciparum* clearance [33].

The cellular production of the pro-inflammatory chemokines MIP3-α/CCL20 and MIG/CXCL9 was inducible by protozoan parasite antigens and mite and yeast allergens, and this wide range of responsiveness to allergens and parasite antigens may augment inflammatory cellular responsiveness. Indeed, the macrophage inflammatory protein MIP3-α/CCL20 and the monokine induced by interferon-gamma MIG/CXCL9 were found elevated in infants with severe malaria [33] and in patients with atopic dermatitis [34,35]. In contrast, helminthes parasites are considered as masterful regulators of specific immune responses [11,36,37], helminth parasite-induced IL-10 may modulate atopic responses in schistosoma-infected children [17], and in filaria-infected mice regulatory T cells will inhibit atopic and airway hyper-reactivity following allergen exposure [18].

Here, in children exposed to and infected with several parasite species not only regulatory IL-10 and anti-inflammatory IL-27 was produced by their PBMC, but also pro-inflammatory IL-33, MIP-3a/CCL20 and MIG/CXCL9 responses were observed, and with an increasing number of parasite infections these pro-inflammatory cytokines and chemokines responses enhanced.

Helminthes infections will active prominent Th2-type cytokines and immune regulatory processes [23] and chronic geo-helminthes infection with high total IgE and anti-*Ascaris* IgG4 may reduce the risk of atopy in school-age children [38], but parasite infections do not in general protect against asthma while hookworm infection may reduce the risk of this disease [39]. In the present study none of the participating children was infected with *Ascaris lumbricoides* and it should be considered that hookworm and *S. haematobium* and *S. mansoni* infections will cause intestinal and urinary tract tissue damage resulting in bloody urines and mostly occult blood in stools; these helminthes will always cause gastrointestinal inflammation. As previously observed, in infants and children with helminth and protozoa infections regulatory cytokine and chemokine responses were not evolved to levels as observed in adults [40], and such immune response profiles may develop with ageing and repeated episodes of exposure and persistent parasite infections. In the present study, allergen-specific positive skin prick test (SPT) responses were more frequent in co-infected children than in infection-free pupils suggesting that parasite co-infections may have triggered or even amplified pro-inflammatory reactivity to allergens.

Furthermore, in the present study we could show that helminthes and protozoan parasite antigen extracts will influence cellular *in vitro* responses to allergens. When PBMC were simultaneously stimulated with *Ascaris lumbricoides* antigens and allergen extracts the production of IL-27, IL-33 and MIP3-α/CCL20 lessened to levels as observed with *Ascaris* antigen exposure alone, suggesting anti-inflammatory effects mediated by the *Ascaris* antigen extract. The potential of *Ascaris* antigen to dampen the cellular production of pro-inflammatory cytokines and chemokines, while regulatory IL-10 remained unaffected, may help to reduce inflammation and hyper reactivity responses. In contrast, the protozoan *Entamoeba* antigen extract enhanced IL-10, lessened the IL-33 release and left IL-27 and MIP3-α/CCL20 production unaffected, again disclosing divergent immune activation patterns to parasite antigen extracts. Thus, the molecular composition of individual parasite species, here helminthes or protozoa, will stimulate distinct cytokine and chemokine responses which may also influence immune responsiveness to allergens.

## Conclusions

In children co-infected with hookworm, schistosomes and intestinal protozoan parasites their PBMC will generate mixed cytokine and chemokine response profiles to parasite antigens and allergens. The molecular composition of individual parasite or allergen extracts will distinctively stimulate regulatory as well as pro-inflammatory chemokines and cytokines, and with an increasing number of parasite infections pro-inflammatory cytokines and chemokines responses enhanced, while regulatory cytokine production remained stable. In poly-parasitized children skin prick test reactivity to allergens extracts was highest suggesting that parasite co-infections may have triggered or amplified their atopic responsiveness.
